# An In Vitro and In Vivo Analysis of the Correlation between Susceptibility-Weighted Imaging Phase Values and R2* in Cirrhotic Livers

**DOI:** 10.1371/journal.pone.0045477

**Published:** 2012-09-18

**Authors:** Ran Tao, Jiuquan Zhang, Yongming Dai, Zhonglan You, Yi Fan, Jinguo Cui, Jian Wang

**Affiliations:** 1 Department of Radiology, Southwest Hospital, Third Military Medical University, Chongqing, China; 2 MR Collaboration NE Asia, Siemens Healthcare, Shanghai, China; 3 Department of Infectious Diseases, Southwest Hospital, Third Military Medical University, Chongqing, China; 4 Department of Radiology, Bethune International Peace Hospital of People’s Liberty Army, Shijiazhuang, Hebei Province, China; Cornell University, United States of America

## Abstract

**Objective:**

To establish a baseline of susceptibility-weighted imaging (SWI) phase value as a means of detecting iron abnormalities in cirrhotic liver and to analyze its relationship with R2*.

**Materials and Methods:**

Sixteen MnCl_2_ phantoms, thirty-seven healthy individuals and 87 cirrhotic patients were performed SWI and multi-echo T2*-weighted imaging, and the signal processing in NMR (SPIN) software was used to measure the radian on SWI phase images and the R2* on T2* maps. The mean minus two times standard deviation (SD) of Siemens Phase Unit (SPU) in healthy individuals was designated as a threshold to separate the regions of interest (ROIs) into high- and low-iron areas in healthy participants and cirrhotic patients. The SWI phase values of high-iron areas were calculated. The R2* values was measured in the same ROI in both healthy participants and patients.

**Results:**

SWI phase values correlated linearly with R2* values in cases of MnCl_2_ concentrations lower than 2.3 mM in vitro (*r* = −0.996, *P*<0.001). The mean value and SD of 37 healthy participants were 2003 and 15 (SPU), respectively. A threshold of 1973 SPU (−0.115 radians) was determined. The SWI phase value and R2* values had a negative correlation in the cirrhotic patients (*r = *−0.742, *P*<0.001). However, no similar relationship was found in the healthy individuals (*r = *0.096, *P* = 0.576). Both SWI phase values and R2* values were found to have significant correlations with serum ferritin concentrations in 42 patients with blood samples (*r* = −0.512, *P = *0.001 and *r = *0.641, *P*<0.001, respectively).

**Conclusion:**

SWI phase values had significant correlations with R2* after the establishment of a baseline on the phase image. SWI phase images may be used for non-invasive quantitative measurement of mild and moderate iron deposition in hepatic cirrhosis in vivo.

## Introduction

There is a significant association between hepatic iron deposition and many chronic hepatic diseases; therefore, hepatic iron deposition could be an important synergistic risk factor for hepatic fibrosis, cirrhosis, and hepatocellular carcinoma [1], [2]. When diagnosing, evaluating and treating hepatic iron deposition, it is important to quantitatively measure the liver iron concentration in vivo. Serum ferritin concentration is the most frequently used indicator of iron overload, but it has poor specificity in the presence of acute inflammation [3], [4]. The gold standard for measuring hepatic iron concentration (HIC) is the liver biopsy. However, the liver biopsy is not often performed because it is an invasive procedure, and repeated biopsies over a short period of time are not feasible in patients with hepatic diseases [5].

Iron is a paramagnetic substance that can shorten the T2 and T2* relaxation time measurements. For this reason, non-invasive magnetic resonance imaging (MRI) methods for the quantitative measurement of HIC have attracted more attention in recent years. Previous MRI methods for non-invasive quantitative measurement have been divided into two categories: 1) measuring the ratio of signal intensities of the liver and paraspinal muscles [6], [7], [8]; and 2) measuring the T2 and T2* values directly through the application of multi-echo gradient echo T2 and T2* sequences, which are converted into reciprocal R2 and R2* values for the quantitative measurement of HIC [9], [10], [11]. The multi-echo T2* scan is the most sensitive of these methods and the most reproducible method for measuring HIC [7], [12].

Susceptibility-weighted imaging (SWI), which exploits the susceptibility differences between tissues as a new type of contrast, can accurately reflect the changes in phase images caused by magnetic substance deposition. SWI phase imaging avoids the impact of main magnetic field inhomogeneity through the implementation of a high-pass filter [13]. Recently, SWI has been used to measure (or infer) the iron content in brain [13], [14]. A recent comparison study between SWI and other image sequences confirmed that SWI can be used as a tool to quantify iron deposition in the brain [15]. Haacke et al. divided the ROIs of different anatomical structures of the brain into high- and low-iron areas (after establishing a baseline) and then measured the phase values of high-iron areas [16]. The results demonstrated it is a sensitive method of evaluating iron content changes. Two-dimensional SWI is a new approach compared with three-dimensional SWI, which is nearly immune to breathing artifacts because it takes advantage of breath-holds. This technique has been successfully applied to the analysis of cirrhotic livers [17].

Our study aimed to establish a baseline with which to quantitatively measure the phase value of high-iron areas in the region of interest (ROI) of cirrhotic liver tissue using a two-dimensional SWI technique and correlated with R2*.

## Materials and Methods

### Phantoms

To validate the relationship between the SWI phase values and R2*, 16 agarose gel phantoms with known concentrations of MnCl_2_ (diameter, 15 mm; volume, 15 ml) were prepared to mimic varying amounts of ferritin (as found in an iron overloaded liver). MnCl_2_ phantom concentrations ranged from 0.1 to 3.1 mM with a 0.2-mM gradient between two adjacent phantoms, which provided R2 values in the range encompassed by healthy through to highly iron-loaded liver [18]. MnCl_2_ was chosen to simulate dispersed iron because its longitudinal relaxation time/transverse relaxation time was comparable to the ratio of aqueous ferritin solutions [19], [20]. Another convenient property of MnCl_2_ is that the atomic weight of Mn (54.9) is close to that of Fe (55.8). Although it is possible to prepare agarose gel phantoms with ferritin, the necessary heating procedure can cause them to be unstable; therefore, MnCl_2_ was utilized to avoid this potential complication.

### Participants

From November 2010 to May 2011, a cohort of 87 patients with hepatic cirrhosis due to chronic type B hepatitis (56 males, 31 females; aged 21 to 64 years; mean age, 47 years) was recruited consecutively. The clinical diagnoses were made based on the patient clinical manifestations, a variety of biochemical markers, and imaging findings [21], [22], [23]. The inclusion criterion was hepatic cirrhosis after chronic type B hepatitis. The exclusion criteria were hepatic cirrhosis from other causes, such as alcoholic cirrhosis, cholestasis cirrhosis, hepatitis C cirrhosis, hereditary metabolic diseases and suspicious benign and malignant tumors.

From April to May 2011, 37 healthy individuals were recruited consecutively for magnetic resonance examination (23 males, 14 females; aged 21 to 60 years; mean age, 45 years). The inclusion criteria were no history of liver diseases, normal liver function, negative results for biochemical markers of hepatitis B, and a negative imaging finding.

The Medical Research Ethics Committee of the Third Military Medical University (Chongqing, China) reviewed and approved the present study. Written informed consent was obtained from each participant prior to the study.

### MRI Data Acquisition

The MRI data were collected using a 3T MRI system (MAGNETOM Trio, Siemens Medical Systems, Erlangen, Germany). The following MR pulse sequences were used for all patients and phantoms: transverse multi-echo T2*-weighted 2D GRE (flip angle of 20°, TR/TE ratio of 226/1.84 to 19.66 ms) and transverse 2D SWI (flip angle of 20°, TR/TE ratio of 150/10 ms, pixel bandwidth of 180 Hz/pixel; WIP#608). For all of the sequences, the FOV was 380×285 mm and the matrix was 384×288; the slice thickness was 5 mm with a gap of 1 mm. The protocol for SWI was similar to that used in a previous study [17]. Three breath-holds were used, each lasting 16 and 21 seconds in SWI and multi-echo T2*-weighted sequences, respectively; 30 slices were used to obtain coverage of the entire liver. The total acquisition time was not longer than 1 minute and 20 seconds, including the break time between the breath holds. After the SWI was performed, the SWI phase images were reconstructed online. The SWI phase images were processed through a 32×32 high-pass filter to remove the low-spatial frequency components [24].

### Radians and R2* Measurements

The data were exported from the MRI scanner to a personal computer, which was used for offline analysis. All of the MRI images were evaluated by the consensus of two experienced radiologists with SPIN (Signal Processing in NMR, Version 1751, MRI Institute for Biomedical Research, Detroit, MI, USA; http://www.wayne.edu/download.htm) software. The largest ROIs were manually outlined in an area that appeared homogeneous and was devoid of vessels in the anterior and posterior segments of the right lobe of the liver at the level of the main portal vein [25]. The areas that were severely affected by artifacts caused by interface of tissue-air, pulsations of the heart and abdominal aorta were not used. The ROIs were first traced on phase images and then copied to the T2* map images, which guaranteed that the boundaries were exactly the same in both images.

The mean and standard deviation (SD) of Siemens Phase Unit (SPU) were obtained from the entire ROIs of healthy participants to calculate a threshold value equal to the mean minus two times the SD (right-handed system) [16]. Based on the threshold, the ROIs of the SWI phase images of the healthy participants and the cirrhotic patients were automatically separated into high- and low-iron areas because an entire ROI analysis would reduce the sensitivity to more subtle changes in iron content when the region containing the iron is a small fraction of the total area [26]. The SPU measurement was performed in the high-iron areas and converted into radians using the following equation: (SPU-2048)×π/2048 [16]. In the same ROIs, the T2* time was calculated and the R2* value was obtained through the 1000/T2* time.

### Measurement of Serum Ferritin in Patients with Hepatic Cirrhosis

Venous blood samples were collected in 42 of the 87 cirrhotic patients to determine the serum ferritin level using a ^125^I radioimmunoassay method with an XH-6020 γ immune counter (The 262 Factory, Xi’an, China) in the Department of Nuclear Medicine, Southwest Hospital, Third Military Medical University.

### Statistical Analysis

SPSS 13.0 software for Windows (SPSS Inc., Chicago, Illinois, USA) was used for statistical analysis. A normality test was performed on the radians measurement of the 37 healthy subjects. The Mann-Whitney U-test was used to compare the differences between the groups. Spearman correlation analysis was used to analyze the relationship between the indicators. A P value of <0.05 was considered statistically significant.

## Results

### Phantoms

**Figure 1:**
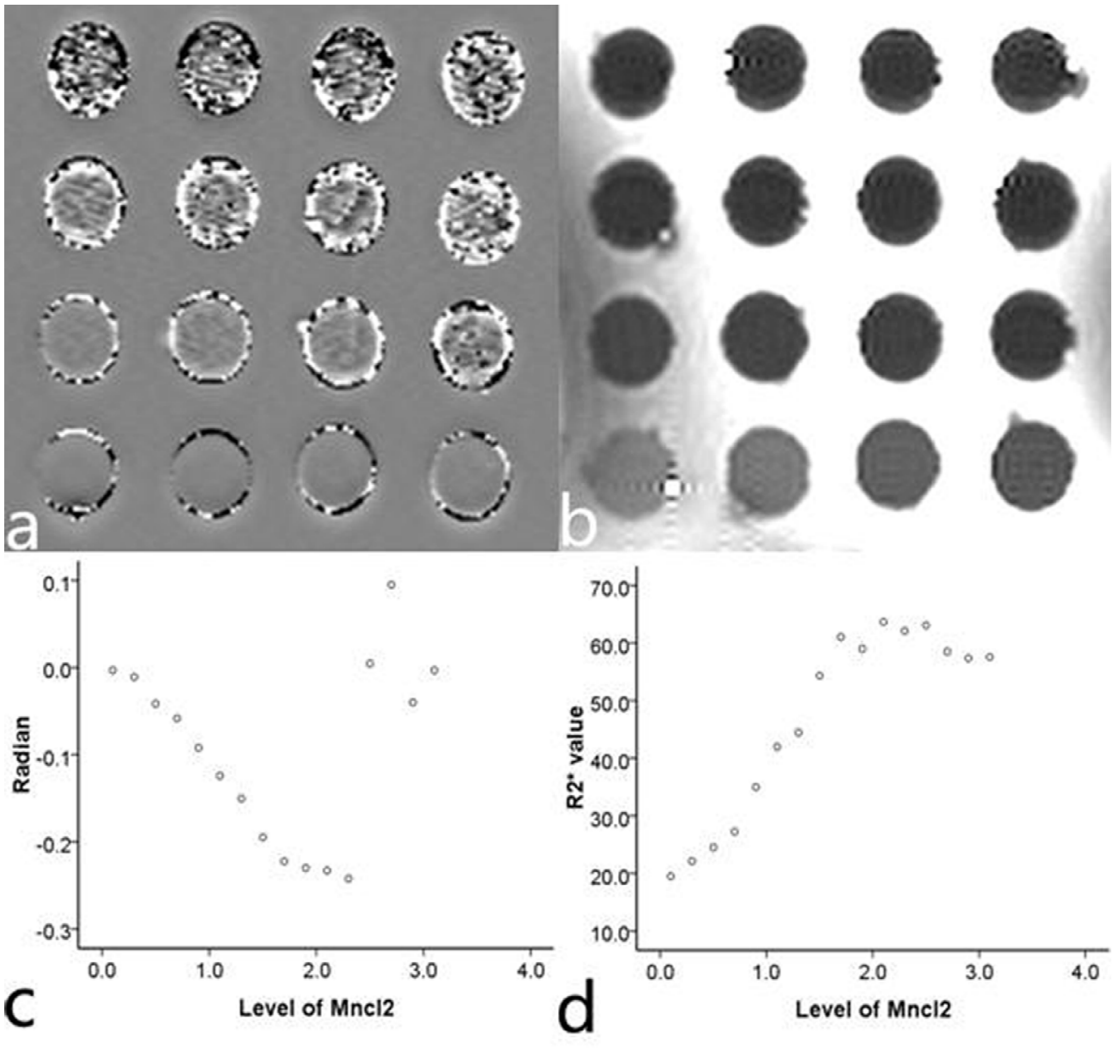
Images of 16 phantoms. This figure shows the SWI phase image (a) and the T2* map image (b) of 16 agarose gel phantoms with various concentrations of MnCl_2_, as well as the correlation of SWI phase values (c) and R2* values (d) with the concentrations of 16 MnCl_2_ phantoms.

**Figure 2:**
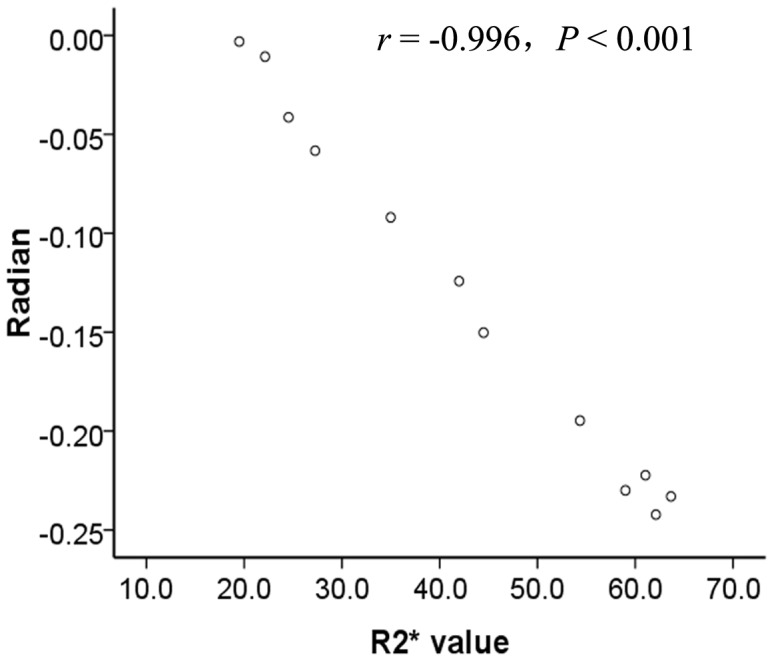
Correlation between the SWI phase values and R2* values in vitro. In cases of MnCl_2_ concentrations lower than 2.3 mM, the SWI phase values correlated linearly with the R2* values (r = −0.996, *P*<0.001).

The SWI phase values and R2* correlated with the level of MnCl_2_ when the phantom concentrations were lower than 2.3 mM; the correlation disappeared when the MnCl_2_ concentration was higher than 2.3 mM (Figure 1). SWI phase values correlated linearly with R2* within the range of 0.1 to 2.3 mM (r = −0.996, *P*<0.001) (Figure 2).

### SPU Measurements in Healthy Subjects

**Figure 3:**
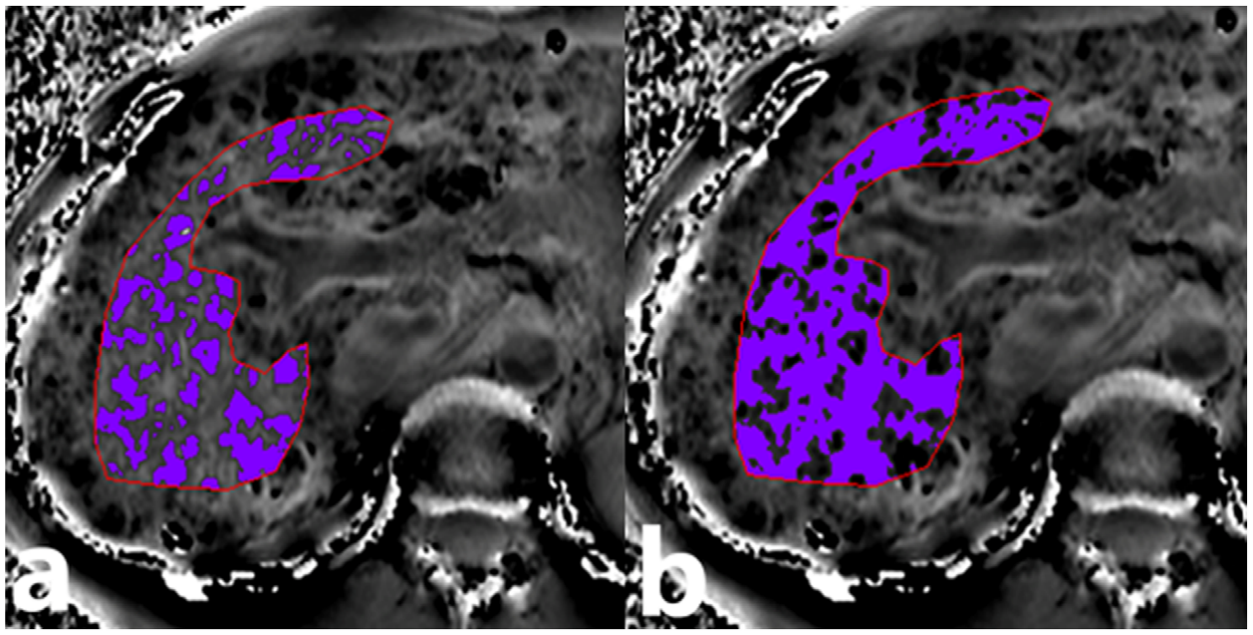
The high- and low-iron areas of SWI phase images. The ROI was divided into a high-iron area (a) and a low-iron area (b) based on the threshold of 1973 SPU.

The SPU measurement obtained from entire ROIs of 37 healthy subjects showed a normal distribution (*Z = *0.676, *P = *0.751). In the 37 healthy subjects, the mean and SD of the SWI phase values were 2003 and 15 (SPU), respectively. Because it was a right-handed system, 1973 SPU (−0.115 radians) was used as the threshold to divide the ROIs of the SWI phase images from the healthy participants and the cirrhotic patients into high- and low-iron areas (Figure 3).

### SWI Phase Values Measurements in High-iron Areas and R2* Values in the ROIs of Healthy Subjects and Cirrhotic Patients

**Figure 4:**
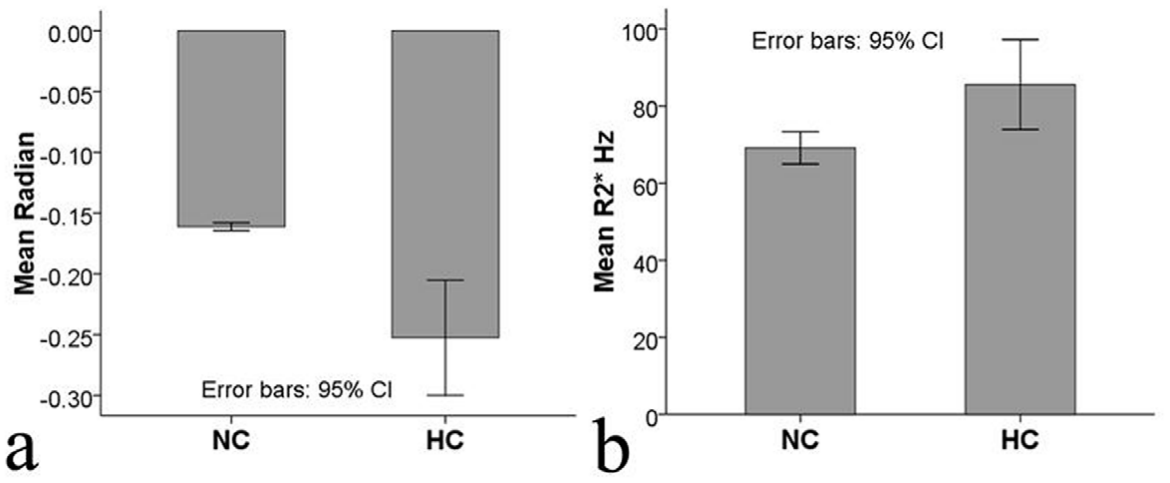
Comparison of the SWI phase values and R2* values in cirrhotic patients and healthy subjects. The SWI phase values (in radians) in high-iron areas of the 37 healthy subjects were significantly higher than those of the 87 cirrhotic patients (−0.161±0.010 vs. −0.266±0.155, respectively; *P*<0.001) (a). The R2* values (Hz) of the 37 healthy subjects were significantly lower than those of the 87 cirrhotic patients (67.02±12.32 vs. 86.30±34.48, respectively; *P = *0.004) (b). The error bar represents the 95% confidence interval. NC: normal control; HC: hepatic cirrhosis patients.

The SWI phase values (in radians) in the high-iron areas of the 37 healthy subjects were significantly higher than those of the 87 cirrhotic patients (−0.161±0.010 vs. −0.266±0.155, respectively; *P*<0.001) (Figure 4a). The R2* values (Hz) of the 37 healthy subjects were also significantly lower than those of the 87 cirrhotic patients (67.02±12.32 vs. 86.30±34.48, respectively; *P = *0.004) (Figure 4b).

### Correlations between SWI Phase Values in High-iron Areas and R2* Values in Cirrhotic Patients and Healthy Subjects

**Figure 5:**
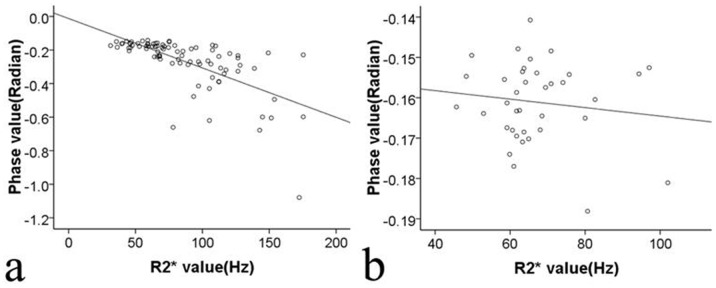
Correlation between the SWI phase values and R2* values in vivo. The Spearman correlation between the SWI phase values and R2* values in 87 cirrhotic patients (*r* = −0.742, *P*<0.001) (a) and in 37 healthy subjects (*r = *0.096, *P* = 0.576) (b).

In the 87 patients with liver cirrhosis, the SWI phase values in the high-iron areas had a negative correlation with R2* values (r = −0.742, *P*<0.001) (Figure 5a). However, in the 37 healthy subjects, the liver SWI phase values in the high-iron areas had no correlation with R2* values (r = 0.096, *P* = 0.576) (Figure 5b).

### Correlations between SWI Phase Values, R2*Values and Serum Ferritin in Cirrhotic Patients

**Figure 6:**
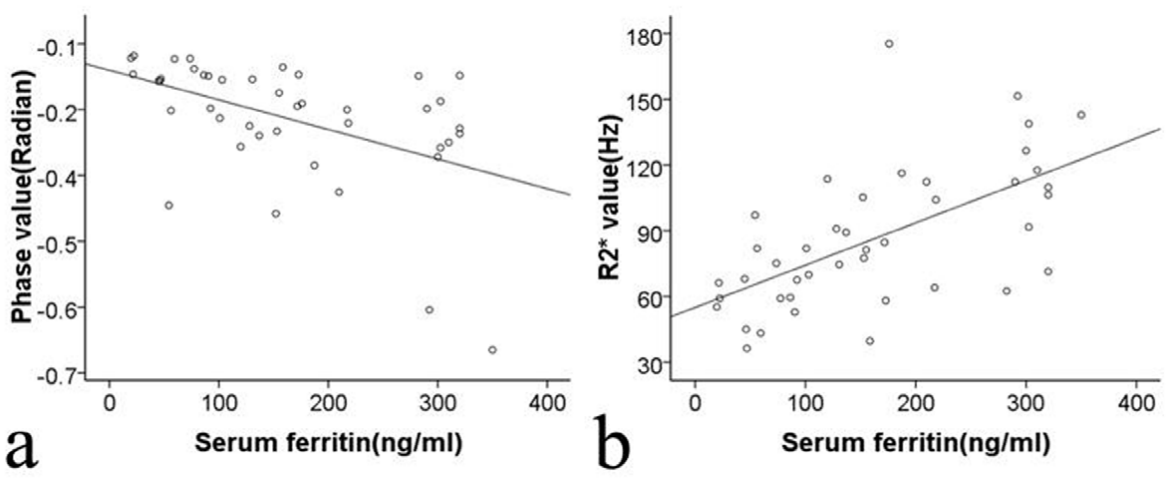
SWI phase values and R2* values correlated with the concentration of serum ferritin. The Spearman correlation between the concentration of serum ferritin and the SWI phase value (*r* = −0.512, *P = *0.001) (a) and between the concentration of serum ferritin and the R2* value (*r = *0.641, *P*<0.001) (b) in 42 cirrhotic patients.

In the 42 cirrhotic patients, the serum ferritin concentrations had a negative correlation with the liver SWI phase values (Figure 6a) and a positive correlation with the R2* values (r = −0.512, *P* = 0.001 and r = 0.641, *P*<0.001, respectively) (Figure 6b).

## Discussion

In the current study, we assessed the relationship between the SWI phase values and R2* values in MnCl_2_ phantoms and in cirrhotic livers. We found that the SWI phase values and R2* values were linearly correlated within the range of 0.1 to 2.3 mM in the MnCl_2_ phantoms and were significantly correlated in cirrhotic livers after the establishment of a baseline.

After establishing a baseline, Haacke et al. [16] divided the ROI of different anatomical structures of the brain into high- and low-iron areas. The purpose was to more sensitively detect iron deposition using SWI phase images and then to compare the SWI phase values with R2* values; there was good correlation between the SWI phase values and R2* values [26]. Theoretically, the breath-hold multi-echo T2*-weighted sequence is the best magnetic resonance method for the measurement of HIC. The method has been shown to have a very good correlation with the results of liver biopsies [7]. We applied the method of Haacke et al. to calculate the SWI phase values in cirrhotic livers and compare them with the R2* values.

The phantoms, which encompassed the range of iron deposition from healthy to highly iron-loaded livers [18], were created for the validation and transferability testing. The results show that both the SWI phase values and the R2* values can be used to measure iron quantitatively in vitro by increasing the concentration of MnCl_2_ up to 2.3 mM. However, the correlation between the SWI phase values and the R2* values disappeared when the MnCl_2_ concentration was greater than 2.3 mM (due to the effect of magnetization saturation). Previous studies demonstrated mild to moderate iron deposition in the livers of patients with cirrhosis [6], [27]; therefore, concentrations from 0.1 to 2.3 mM could encompass the range of iron deposition in these patients.

In vivo, HIC increases in cirrhotic patients [28]. It is already well known that phase, which reflects field inhomogeneity, is the results of the tissue susceptibility convolved with a dipole kernel. Being paramagnetic, iron can cause inhomogeneity of the local magnetic fields and change the SWI phase values and R2* values. To some extent, the more iron that is in the tissue, the more the phase will decrease [16]. In T2*WI, the T2* time results from a combination of the effects of the spin-spin relaxation (T2) and the relaxation (T2′) caused by the inhomogeneity of the magnetic field. In a cirrhotic liver, the T2′ was caused by iron deposition and the dephasing was caused by the inhomogeneity of the magnetic field, chemical shift, and magnetic field gradients resulting from spatial encoding [29]. With the increased concentration of iron in the livers of cirrhotic patients, the shortening of the T2* relaxation time is mainly determined by iron deposition. Due to the above reasons, the SWI phase values (in radians) in the 87 cirrhotic patients demonstrated a good correlation with the R2* values, and both correlated well with the level of serum ferritin. Excessive iron deposition in the liver tissue will exceed the ferritin reserve of the liver cells and lead to increased serum ferritin [4]; therefore, ferritin levels have traditionally been used as an indicator of iron overload in the liver. However, a coincidental acute inflammatory event may increase the serum ferritin concentration measured at the time of MRI without increasing the level of hepatic iron [3], [4].

The results showed no correlation between the SWI phase values and R2* values in the 37 healthy subjects. This was likely due to the difference in the mechanisms associated with the SWI phase and the T2*WI imaging. Theoretically, the T2* effect occurs during dephasing due to the inhomogeneity of the magnetic field spreads among the voxels. It must be remembered that signal dephasing occurs because a phase spread exists across a voxel. Without phase dispersion, there is no extra T2* effect. Therefore, tissues that have very low and uniform iron distributions will exhibit a phase effect but no T2* effect [30]. When the iron concentration reaches certain levels in tissue, the dephasing effect can occur among the voxels, and the T2* time is reduced. With the increasing spatial resolution of the gradient echo sequence scan (the voxel size of the T2* scan sequence was 0.9×0.9 mm in this study), the spread of dephasing among voxels tends to disappear. Furthermore, when the iron concentration is low and homogeneously distributed, which is not enough to produce the dephasing effect among the voxels, the T2* effect caused by iron deposition disappears [30], [31].

Subtle magnetic changes within a tissue can lead to changes in SWI phase values. However, when the concentration of iron increases to a certain level, the correlation disappears. Aliasing occurs because the SWI phase value rotates by more than 2π. This problem can be avoided by using phase-unwrapping algorithms [32] and a multiple-echo SWI sequence [33], [34].

SWI phase images are not directly dependent on T1 and T2 relaxation parameters and are less affected by the increased inhomogeneity of radio frequency magnetic fields at high main magnetic field strengths, which means that it could be used for the quantitative measurement of tissue properties [31]. However, there are many other factors that affect the phase in vivo, e.g., the inhomogeneity of the main magnetic field, the echo time used in the gradient-echo imaging sequence, the main magnetic field strength, the tissue orientation relative to the main magnetic field, the geometry of the structures involved, and the high-pass filter kernel size used in phase image processing. These factors should be accounted for in the quantitative measurement of tissue properties using SWI phase images (although some have been eliminated through the implementation of proper procedures).

This study has several limitations. First, in addition to iron, the liver normally contains other essential elements that can contribute to phase changes, such as calcium [35], copper, and zinc. We cannot rule out the possibility that in some small regions of the liver, the concentration of calcium or copper might be sufficient to induce detectable phase shifts unrelated to those caused by iron. Second, the phase values were obtained from the ROI in a single slice, which may not indicate iron deposition in the liver. Third, the reliability of the threshold (−0.115 radians), which was used as a baseline to measure the liver SWI phase values, needs to be confirmed with a larger sample size and with scanners from other manufacturers.

### Conclusions

The results of the study showed that SWI phase values correlated with R2* values in vitro (within the range of 0.1 to 2.3 mM) and in vivo. Both methods can non-invasively quantify the measurement of liver iron concentrations in patients with cirrhosis after type B hepatitis. Due to the difference of the imaging principle and the varying sensitivity to iron concentrations (of the SWI phase and R2* values), further studies measuring liver iron concentrations by liver biopsy are needed to confirm these results.
